# Supplementary material for: Orthodontic Treatment Does Not Affect Frontal Sinus Development in Female Adults: A Clinical Study
Source: J Clin Med. 2023 Jan 18;12(3):778. doi: 10.3390/jcm12030778 (PMC9917380; doi:10.3390/jcm12030778)
Supplement: Supplementary file 1 [file jcm-12-00778-s001.zip › jcm-2112424-supplementary.pdf]

Table S1. Changes in cephalometric measurements during orthodontic treatment and a simple linear regression analysis for the measurement values.

Angular measurement items

		Orthodontic treatment		*p*-value		
		Before	After	Interaction	Classification	Time
SNA angle	Average angle	81.0 ± 4.4	80.8 ± 4.1			
	High angle	79.4 ± 2.8	77.5 ± 2.9	0.019	0.045	0.001
	Low angle	80.9 ± 3.2	80.4 ± 2.9			
	Whole	80.4 ± 3.6	79.5 ± 3.6			
SNB angle	Average angle	77.3 ± 2.7	77.1 ± 3.0			
	High angle	72.7 ± 3.3	71.9 ± 3.0	0.350	0.000	0.014
	Low angle	78.5 ± 3.1	78.2 ± 3.4			
	Whole	76.1 ± 3.9	75.6 ± 4.2			
ANB angle	Average angle	3.7 ± 3.2	3.7 ± 2.7			
	High angle	6.7 ± 1.9	5.7 ± 1.5	0.038	0.000	0.028
	Low angle	2.4 ± 3.0	2.3 ± 2.2			
	Whole	4.3 ± 3.2	3.9 ± 2.6			
Facial angle	Average angle	86.4 ± 2.5	87.2 ± 2.5			
	High angle	81.0 ± 3.1	81.1 ± 2.9	0.525	0.000	0.110
	Low angle	88.7 ± 2.6	89.0 ± 2.3			
	Whole	85.2 ± 4.2	85.6 ± 4.3			
Y-axis	Average angle	63.2 ± 2.3	62.7 ± 2.1			
	High angle	70.0 ± 3.2	69.8 ± 3.2	0.680	0.000	0.307
	Low angle	60.5 ± 2.0	60.4 ± 2.2			
	Whole	64.7 ± 4.8	64.4 ± 4.8			
Occ.lusal plane angle	Average angle	89.4 ± 4.9	90.3 ± 3.3			
	High angle	86.7 ± 6.6	89.5 ± 3.6	0.286	0.090	0.003
	Low angle	91.0 ± 7.2	92.4 ± 5.5			
	Whole	89.0 ± 6.4	90.7 ± 4.3			
Gonial angle	Average angle	123.1 ± 5.8	124.5 ± 5.9			
	High angle	131.0 ± 7.0	130.4 ± 7.7	0.041	0.000	0.027
	Low angle	114.6 ± 5.4	116.7 ± 4.7			
	Whole	123.1 ± 9.0	124.1 ± 8.3			
FMA	Average angle	28.6 ± 1.8	28.9 ± 1.8			
	High angle	40.3 ± 2.8	39.5 ± 2.8	0.059	0.000	0.677
	Low angle	20.4 ± 1.9	21.2 ± 2.6			
	Whole	30.1 ± 8.5	30.2 ± 7.9			
Palatal plane angle	Average angle	7.3 ± 3.6	8.79 ± 4.2			
	High angle	11.7 ± 3.2	13.35 ± 4.2	0.993	0.000	0.001
	Low angle	7.6 ± 3.0	9.21 ± 2.7			
	Whole	8.9 ± 3.8	10.49 ± 4.2			

U1 to SN	Average angle	107.8 ± 10.3	100.7 ± 8.1			
	High angle	104.2 ± 7.0	95.4 ± 10.3	0.218	0.058	0.000
	Low angle	106.1 ± 11.3	103.8 ± 7.9			
	Whole	106.0 ± 9.6	99.8 ± 9.4			
Interincisal angle	Average angle	120.5 ± 11.4	130.1 ± 11.1			
	High angle	112.7 ± 9.6	127.9 ± 10.7	0.036	0.091	0.000
	Low angle	124.1 ± 14.2	125.9 ± 9.2			
	Whole	118.9 ± 12.6	128.0 ± 10.4			
IMPA	Average angle	94.6 ± 7.4	91.2 ± 9.7			
	High angle	93.8 ± 5.2	87.7 ± 7.6	0.222	0.000	0.004
	Low angle	100.3 ± 6.8	99.3 ± 9.5			
	Whole	96.1 ± 7.0	92.5 ± 10.0			
FMIA	Average angle	56.8 ± 6.8	59.9 ± 9.8			
	High angle	46.0 ± 5.8	52.8 ± 7.4	0.087	0.000	0.006
	Low angle	59.4 ± 6.6	59.6 ± 8.7			
	Whole	53.9 ± 8.6	57.4 ± 9.2			

Linear measurement items

		Orthodontic treatment		*p*-value		
		Before	After	Interaction	Classification	Time
SN	Average angle	69.7 ± 2.9	69.7 ± 2.9			
	High angle	68.6 ± 2.3	68.8 ± 2.5	0.394	0.537	0.339
	Low angle	69.1 ± 3.6	69.1 ± 3.6			
	Whole	69.1 ± 2.9	69.2 ± 3.0			
Overjet	Average angle	5.5 ± 2.6	3.7 ± 0.6			
	High angle	5.8 ± 3.5	3.6 ± 1.0	0.323	0.105	0.001
	Low angle	3.9 ± 3.5	3.2 ± 0.9			
	Whole	5.1 ± 3.3	3.5 ± 0.8			
Overbite	Average angle	2.8 ± 1.7	2.3 ± 1.0			
	High angle	0.6 ± 2.3	2.0 ± 0.8	0.002	0.012	0.873
	Low angle	2.5 ± 2.5	1.7 ± 0.9			
	Whole	2.0 ± 2.3	2.0 ± 0.9			
Wits appraisal	Average angle	0.3 ± 3.6	-0.3 ± 2.5			
	High angle	2.6 ± 5.4	0.4 ± 2.9	0.260	0.075	0.008
	Low angle	-0.9 ± 5.3	-1.7 ± 3.9			
	Whole	0.7 ± 4.9	-0.5 ± 3.2			
N-Me	Average angle	127.0 ± 3.2	126.9 ± 3.2			
	High angle	134.0 ± 4.4	133.7 ± 4.2	0.059	0.000	0.803
	Low angle	125.3 ± 3.3	125.5 ± 3.1			
	Whole	128.9 ± 5.3	128.8 ± 5.0			
Ar-Go	Average angle	50.2 ± 4.2	50.2 ± 4.2			
	High angle	45.5 ± 4.2	45.7 ± 4.3	0.394	0.000	0.339

	Low angle	54.0 ± 3.8	54.0 ± 3.8			
	Whole	49.7 ± 5.3	49.8 ± 5.3			
Ar-Me	Average angle	108.6 ± 4.4	108.6 ± 4.4			
	High angle	103.4 ± 5.0	103.4 ± 5.1	0.839	0.000	0.121
	Low angle	109.4 ± 3.7	109.4 ± 3.7			
	Whole	107.0 ± 5.1	107.1 ± 5.1			
Go-Me	Average angle	71.5 ± 4.5	71.5 ± 4.5			
	High angle	67.8 ± 4.0	67.5 ± 3.8	0.338	0.002	0.433
	Low angle	72.0 ± 3.6	72.0 ± 3.6			
	Whole	70.3 ± 4.4	70.3 ± 4.4			

Red text indicates variables with statistically significant differences between the pre-treatment and post-treatment values and among three subgroups. Low angle, low mandibular plane angle group; Average angle, average mandibular plane angle group; High angle, high mandibular plane angle group
